# Anesthetic management with remimazolam in a patient with Child-Pugh C liver cirrhosis: a case report

**DOI:** 10.1186/s40981-022-00590-9

**Published:** 2022-12-27

**Authors:** Satoshi Uchida, Daiki Takekawa, Eiji Hashiba, Reiko Kudo, Kazuyoshi Hirota

**Affiliations:** 1 Department of Anesthesiology, Hirosaki University Graduate School of Medicine, 5 Zaifu-cho, Hirosaki, 036-8562 Japan; 2 Division of Intensive Care, Hirosaki University Hospital, 53 Hon-cho, Hirosaki, 036-8562 Japan

**Keywords:** Remimazolam, Flumazenil, Liver cirrhosis, Child-Pugh score

## Abstract

**Background:**

Remimazolam is a new ultra-short-acting benzodiazepine, and its sedative effect is prolonged in patients with hepatic impairment. This is the first report of remimazolam anesthesia in a patient with Child-Pugh C liver cirrhosis.

**Case presentation:**

A 52-year-old female was diagnosed with tongue cancer and scheduled for partial glossectomy. Preoperative examinations revealed Child-Pugh C liver cirrhosis, but the pathogenesis was unknown. We scheduled remimazolam anesthesia because it would stabilize her intraoperative circulation. We managed with a much lower-than-normal dose of remimazolam; even so, the patient required flumazenil to regain consciousness. She was admitted to the intensive care unit, but her consciousness remained clear even after the effect of flumazenil had worn off.

**Conclusion:**

We experienced anesthetic management with remimazolam in a patient with Child-Pugh C liver cirrhosis. Even conservative use of remimazolam in patients with severe hepatic dysfunction may result in emergence times that are delayed longer than expected.

## Background

Remimazolam is a new ultra-short-acting benzodiazepine. Its effect is prolonged among patients with severe hepatic impairment because remimazolam is metabolized in the liver [1]. The drug was designed to be rapidly metabolized by a variety of tissue esterases. Thus, some non-hepatic metabolism of remimazolam occurs in humans [2], but it remains unclear to what degree these other tissues contribute. Furthermore, the interaction between remimazolam anesthesia and hepatic function has not yet been elucidated, and the dosage of remimazolam for patients with severe hepatic dysfunction has not been established.

There is one previous report about successful anesthetic management with remimazolam in a patient with Child-Pugh B liver cirrhosis [3], but our PubMed search revealed no reports of remimazolam in a Child-Pugh C case. Herein, we report anesthetic management using remimazolam in a patient with Child-Pugh C (score 10) liver cirrhosis. The aim of this report was to review the indications for and potential effects of remimazolam use in a patient with severe hepatic impairment.

## Case presentation

We obtained written informed consent from the patient for the publication of this case report. A 52-year-old female [height, 158 cm; weight, 89.9 kg; body mass index (BMI), 35.6 kg/m^2^] was diagnosed with tongue cancer and scheduled for partial glossectomy. Preoperative examination revealed Child-Pugh C (score 10) liver cirrhosis, but the pathogenesis was unknown despite a detailed examination by gastroenterologists. A blood test showed elevated bilirubin (3.0 mg/dL; normal, 0.3–1 mg/dL), decreased albumin (2.7 g/dL; normal, 3.4–5.4 g/dL), decreased prothrombin index (53%; normal, 85–100%), low platelet count (44,000/μL; normal, 150,000–450,000/μL), and low fibrinogen level (133 mg/dL; normal, 200–400 mg/dL). Abdominal computed tomography showed atrophied liver, mild ascites, esophageal varices, and splenomegaly. No evidence of encephalopathy was observed, but the serum ammonia level was elevated at 188 μg/dL (15–45 μg/dL). Based on these examination results, the patient was diagnosed with decompensated liver cirrhosis. The patient had received a transfusion of 10 units of concentrated platelet product the day before surgery.

**Fig. 1:**
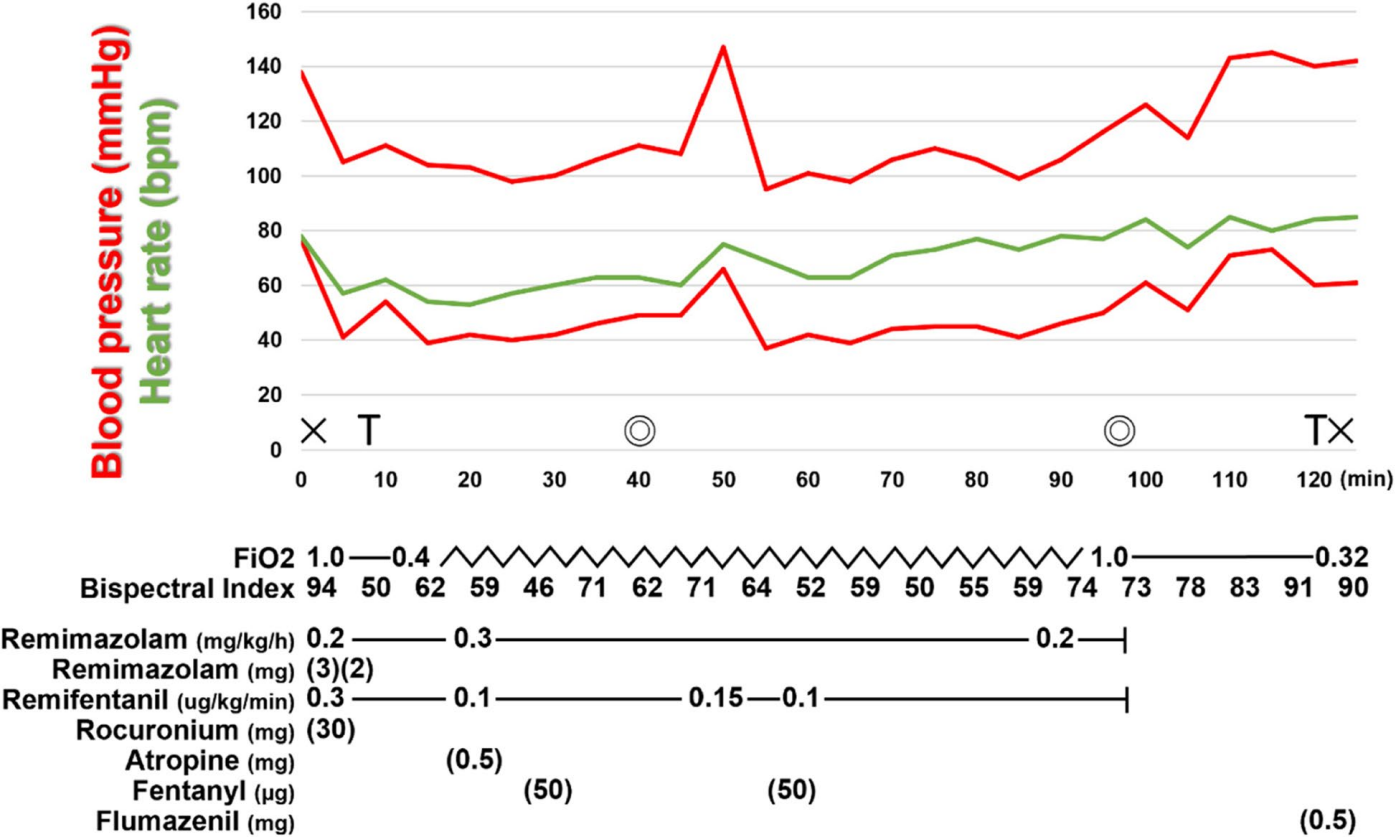
Time course of general anesthesia. During general anesthesia, the patient’s hemodynamics were stable and neither hypotension nor tachycardia was observed. Spontaneous respiration appeared shortly after the end of remimazolam administration, but the patient was unable to follow the indicated actions due to a lack of arousal. The patient’s level of consciousness did not improve even after 25 min had passed. The patient awoke promptly with 0.5 mg of flumazenil, allowing tracheal extubation. ×, the start and end of general anesthesia; T, tracheal intubation and extubation; ◎, the start and end of surgery

The patient’s vital signs upon entering the operating room were as follows: blood pressure, 147/76 mmHg; heart rate, 81 bpm; SpO2, 98% under room air. General anesthesia was induced with 7 mg (0.08 mg/kg) of remimazolam, 0.3 μg/kg/min of remifentanil, and 30 mg of rocuronium. We successfully intubated the trachea using a McGRATH™ MAC video laryngoscope (McGRATH; Aircraft Medical Ltd., Edinburgh, UK) under stable vital signs. General anesthesia was maintained with 0.2–0.3 mg/kg/h of remimazolam with reference to the bispectral index and 0.1–0.15 μg/kg/min of remifentanil. The resulting intraoperative circulation was stable; thus, we used no vasopressor agents (Fig. 1). The depth of anesthesia was monitored using the bispectral index and remained in the range of 50–70. There was no intraoperative body movement and no intraoperative arousal. The surgery lasted 55 min, with 70 g of total blood loss. Intraoperatively, 4 units of fresh frozen plasma and 10 units of concentrated platelet product were transfused.

The patient resumed spontaneous breathing soon after the end of remimazolam administration, but after 25 min, she still had not awoken. The train-of-four ratio, which registers the neuromuscular blockade, had already recovered to 95%. The patient awoke promptly with 0.5 mg of flumazenil, allowing tracheal extubation. The duration of anesthesia was 2 h 5 min, and the total dose of remimazolam delivered was 48 mg. The patient was transferred to the intensive care unit (ICU), but her level of consciousness did not decrease again. We found no worsening of her liver function during the perioperative period. After the surgery, she was transferred to the department of gastroenterology for treatment of her cirrhosis. She was discharged 14 days after the operation without any complications.

## Discussion

We experienced anesthetic management with remimazolam in a patient with Child-Pugh C (score 10) liver cirrhosis. There is one previous report of remimazolam anesthesia for a patient with Child-Pugh B (score 7) liver cirrhosis undergoing laparoscopic cholecystectomy [3]. In that report, remimazolam was administered at 0.5–0.8 mg/kg/h during the operation despite its normal maintenance dose being 1 mg/kg/h; indeed, the remimazolam drug package insert suggests a cautious titration approach in patients with moderate to severe hepatic dysfunction. Rapid awakening was achieved without flumazenil in that case. In our Child-Pugh C case, we maintained general anesthesia with only 0.2–0.3 mg/kg/h of remimazolam. Although this was a very low dosage compared to the normal dosage, the dosage was determined based on actual weight rather than ideal weight, which may have resulted in a relative increase in dosage due to severe obesity. The dose of remimazolam was adjusted with reference to the BIS value, but the possibility that the BIS value was calculated lower due to hyperammonemia cannot be ruled out [4]. Even so, the patient requires reversal with flumazenil to be awakened despite having regained spontaneous respiration.

We had planned to administer remimazolam for this liver cirrhosis case because it has several advantages compared to other anesthetics from the viewpoint of circulatory maintenance and airway management. Remimazolam is less likely to cause hypotension compared to other anesthetics [5], making it particularly useful for patients prone to circulatory instability. Liver cirrhosis is known to be one of the conditions that predisposes patients to circulatory collapse during anesthesia, likely due to lower circulating plasma volume because of decreased albumin production [6]. Remimazolam anesthesia has the advantage of maintaining circulatory dynamics, thus avoiding a decrease in hepatic blood flow. Another concern was the patient’s obesity, at a BMI of 35.6 kg/m^2^, making airway management challenging; as remimazolam is antagonized rapidly by flumazenil, using remimazolam for anesthetic induction decreases the risk of “cannot intubate, cannot ventilate.” In addition, since this case was a cancer surgery, we planned total intravenous anesthesia because it is recurring reported that inhalational anesthetics are potent immunosuppressive and tumorigenic agents [7]. Also, hepatotoxicity of halogenated inhalational anesthetics has been well known [8], but there are no reports of hepatotoxicity caused by remimazolam. On the contrary, remimazolam reduced the liver injury and pathological changes in rat models because of its anti-inflammatory effects [9].

Given these issues, remimazolam anesthesia was thought to be suitable for our case, but we were nonetheless concerned about delayed emergence due to the accumulation of unmetabolized drug. The only metabolizing enzyme of remimazolam, carboxylesterase, was reported to have 20–40% decreased activity in an ex vivo cirrhotic model [10]. Furthermore, the clearance of remimazolam is 38.1% lower for patients with Child-Pugh score ≥ 10 [5]. Decreased clearance leads to increased drug blood concentrations because these are inversely related. When remimazolam is administered to patients with severe hepatic impairment, drug concentrations may be elevated even at reduced doses. In addition, the protein binding of remimazolam is very high, and hypoalbuminemia increases free drug. Therefore, the anesthetic effect may be enhanced in patients with decreased protein production, as in this case.

However, there may be other factors besides hepatic impairment that prolong the remimazolam effect. Decrement time for remimazolam plasma concentration increases as the duration of continuous infusion exceeds 30 min [11], but in this case, remimazolam was administered for more than 3 h. In addition, a higher BMI prolongs the time to extubation in remimazolam anesthesia [12], and this patient had a BMI of 35.6 kg/m^2^. This may be due to the accumulation of remimazolam in the peripheral tissues. Although there were no perioperative findings suggestive of hepatic encephalopathy, ammonia and anesthetics have a synergistic effect, and hyperammonemia affected on the arousal.

It is generally known that when patients with severe liver impairment are administered flumazenil as an antagonist of midazolam, they should be closely monitored after awakening because a sedative effect can sometimes reappear. This phenomenon, called re-sleeping, has also been reported with the combination of remimazolam and flumazenil, requiring a second dose of 0.5 mg flumazenil in the ward 45 min after first antagonizing [13]. As our patient had a severe hepatic impairment, there was a concern about re-sleeping, and we continued close observation of the patient’s postoperative consciousness in the ICU. Fortunately, she maintained consciousness. Re-sleeping might have been less likely to occur in this case because flumazenil was not administered until 25 min after cessation of remimazolam. In cases in which flumazenil is administered immediately after cessation of remimazolam in patients with severe hepatic impairment, clinicians should take special precautions for the possibility of re-sleeping.

## Conclusion

We managed general anesthesia for a Child-Pugh C liver cirrhosis patient with a remimazolam-based anesthetic. We administered a low maintenance infusion rate of remimazolam based on the bispectral index value. Although the patient required reversal with flumazenil, the presence of an antagonist is an advantage of remimazolam as a new anesthetic. It is not important to avoid its use and wait for awakening, but rather to administer it appropriately and monitor the patient closely after surgery. However, clinicians should be aware that even with reduced dosing of remimazolam in patients with severe hepatic dysfunction there may be emergence times that are much longer than expected.

## Data Availability

Not applicable.

## Abbreviations

BMI: Body mass index
ICU: Intensive care unit
